# The Rooibos Benefit Sharing Agreement–Breaking New Ground with Respect, Honesty, Fairness, and Care

**DOI:** 10.1017/S0963180119001075

**Published:** 2019-11-05

**Authors:** DORIS SCHROEDER, ROGER CHENNELLS, COLLIN LOUW, LEANA SNYDERS, TIMOTHY HODGES

**Keywords:** Benefit sharing, Convention on Biodiversity, Nagoya Protocol, San People, San Code of Research Ethics, Rooibos

## Abstract

The 1992 Convention on Biological Diversity (CBD) and its 2010 Nagoya Protocol brought about a breakthrough in global policy making. They combined a concern for the environment with a commitment to resolving longstanding human injustices regarding access to, and use of biological resources. In particular, the traditional knowledge of indigenous communities was no longer going to be exploited without fair benefit sharing. Yet, for 25 years after the adoption of the CBD, there were no major benefit sharing agreements that led to significant funding streams for indigenous communities. This changed with the signing of the Rooibos Benefit Sharing Agreement in South Africa, described in this paper. As the authors report, the Rooibos Agreement is a superlative in two respects. It is the biggest benefit sharing agreement between industry and indigenous peoples to date. It is also the first *industry-wide* agreement to be formed in accordance with biodiversity legislation. This article is a co-production between traditional knowledge holders, the lawyer who represented their interests, the Co-Chair of the Nagoya Protocol negotiations, and an ethicist who analyzed the major challenges of this historic agreement. With no precedent in the benefit sharing world, the agreement stands as a concrete example of the ‘art of the possible.’ Although the rooibos case is unique in a number of aspects, the experience offers many transferable insights, including: patience; incrementalism; honesty; trust; genuine dialogue; strong legal support; a shared recognition that a fair, win-win deal is possible; government leadership; and unity amongst indigenous peoples. Such ingredients of success can apply well beyond southern Africa.

## Introduction

The 1992 Convention on Biological Diversity^1^ (CBD) was a breakthrough in global policy making. It combined a concern for the environment with a commitment to resolving longstanding human injustices regarding access to and use of biological resources.^2^ With the exception of the United States, all countries on earth have ratified the CBD, which aims to conserve biodiversity, achieve its sustainable use, and reward its custodians with fair and equitable benefit sharing.^2^

The CBD was agreed more than a quarter-century ago. The 2010 Nagoya Protocol,^3^ which supplements the CBD with specific legally-binding obligations on benefit sharing from the use of genetic resources and associated traditional knowledge (TK), celebrates its 10^th^ birthday in 2020. The 2004 South African National Environmental Management Biodiversity Act^4^ is 15 years old. All of the above require that benefits from the use of biological resources are shared with the holders of TK, in particular indigenous communities.^5^

Yet, if one searches academic databases, the grey literature, and global search engines for successful access and benefit sharing (ABS) agreements with indigenous communities, one finds almost nothing. Most information gives advice on how to negotiate a successful ABS agreement,^6^ or delivers sample contracts without substantial examples.^7^ One could almost say that the world is still waiting for the first big, successful, monetized ABS agreement associated with TK.

Initially, hopes were high that the San community of Southern Africa was going to be the first indigenous group to be rewarded with a regular income stream for guarding TK. The San are said to be the oldest genetic ancestors of modern humans.^8^ They hold knowledge on a wide range of indigenous southern African plants.^9^ Known for their distinctive click languages, San numbers have now dwindled to approximately 111,000 people living primarily in Botswana, Namibia and South Africa, with small remnant populations in Angola, Zimbabwe, Zambia and Mozambique.

The first benefit sharing agreement negotiated by the South African San related to the appetite- and thirst-suppressant properties of the hoodia succulent. Signed by San representatives and a South African research institute (Council for Scientific and Industrial Research, CSIR) in 2003, the agreement allowed the CSIR to profit legally and ethically from an earlier patent on Hoodia properties by licensing their patent,^10^ first to Pfizer then to Unilever (Wynberg, Schroeder, Chennells 2009). Whilst milestone payments were made into the San Hoodia Trust, no commercial product was ever developed, and both Pfizer and Unilever returned their licenses to the CSIR. For a second San benefit sharing agreement in 2008, which has received little international attention despite having a successful product associated with it, see Box 1.^11^

Box 1. The Zembrin^®^ Benefit Sharing Agreement

In 2008, the South African San Council signed its second benefit sharing agreement, covering traditional knowledge of the sceletium plant. Standardized botanical extracts of the plant are now used in a product called Zembrin^®^ to counter anxiety, stress, and depression. The patent-protected active components of the plant are currently marketed by HG&H Pharmaceuticals in South Africa, the USA, Canada, Brazil, Malaysia, and Japan. 5 percent of all sales of the extract are paid into a trust fund for the San peoples, with a further 1 percent paid for the use of a San logo on the product. The proceeds are shared equally with two communities in Namaqualand, who provided a major lead to the commercial developer.

The third major San and Khoi benefit sharing agreement is likely to be a big occasion. On 25 March 2019, the San, the Khoi^12^ and the South African rooibos industry signed the Rooibos Benefit Sharing Agreement (RBSA), which is the first comprehensive, industry-wide benefit sharing agreement and globally without parallel. It is exceptional as it not only spans an entire industry, but also because the product is already on the market. Hence, there is no translation gap.^13^

This article analyzes the following aspects of the Rooibos agreement:

• Why traditional knowledge about the rooibos plant is subject to a benefit sharing agreement.
• The process followed from claiming that benefit sharing was due, through to signing an agreement.
• The details of the Rooibos Agreement.
• The main challenges: Why did the process take nine years?
• How the agreement was influenced by the 2017 San Code of Research Ethics^14^ and in particular, the values of respect, honesty, fairness, and care.
• The policy implications of the Rooibos Agreement, and its potential to influence benefit sharing agreements involving indigenous communities in other regions of the world.

## Rooibos

**Figure 1.**
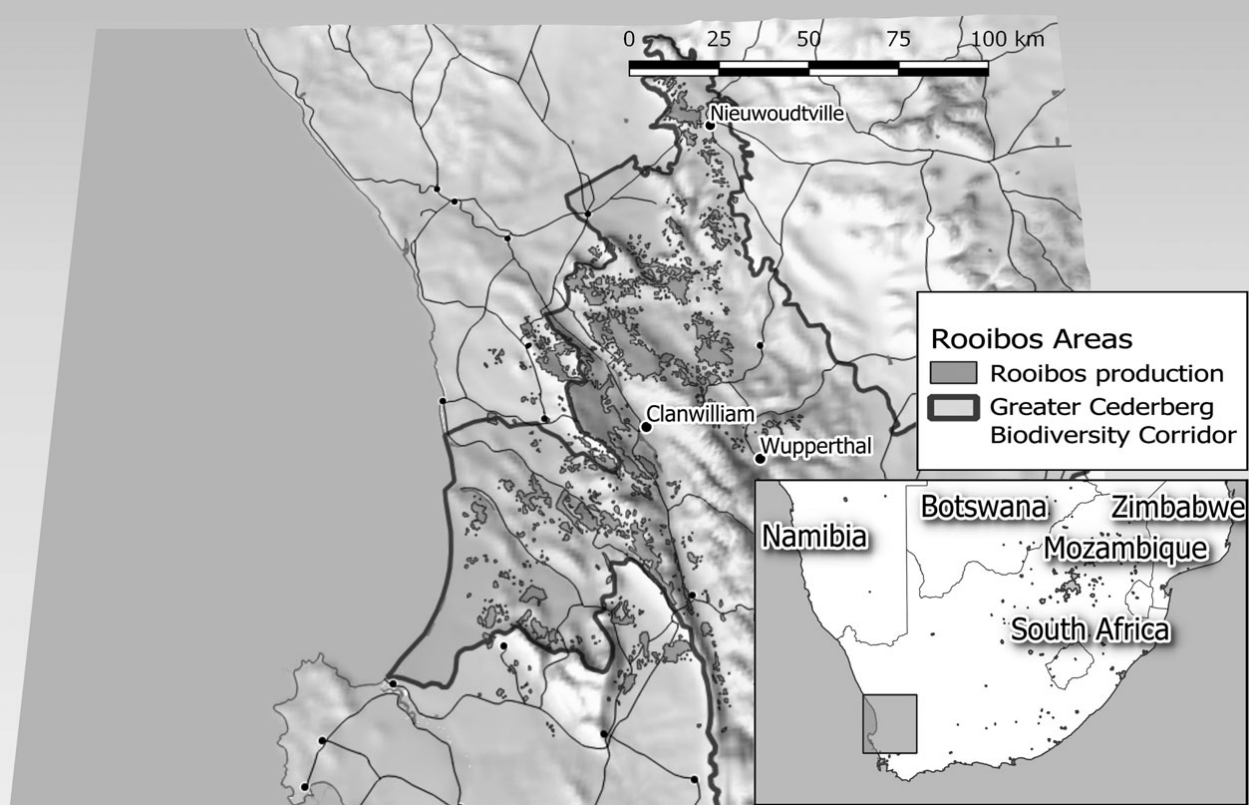
Production areas of rooibos in the Cederberg Mountains (*figure based on three earlier diagrams).* ^19^

Rooibos or *Aspalanthus Linearis* is a plant primarily known around the world as a tea.^15^ Its history of commercialization dates back at least 150 years.^16^ Today, the rooibos tea industry employs 5,000 people in South Africa, produces around 15,000 tons of processed leaves per year, of which half are exported, and generates an income of approximately 500 million Rand per year (29 million Euros).^17^ In 2014, rooibos received geographical indication status, the first nonalcoholic South African product to be so designated.^18^ As a result, only rooibos tea from the indicated area (the Cederberg mountains of South Africa, see Figure 1) can legitimately be called rooibos.

Given that rooibos is known primarily as a tea, one might ask why it should even be considered for benefit sharing under the CBD. Is benefit sharing not restricted to research and patent-related developments, as in the hoodia case?

This was the question raised by representatives of the rooibos industry in response to the TK holder request to enter benefit sharing negotiations under NEMBA, the South African Biodiversity Act. The industry’s question had two main aspects:

1. Why should there be a legal basis for restitution claims for traditional knowledge that was given by Khoi and San ancestors to European settlers in the 18th century? The CBD was only agreed in 1992 (and is not considered retroactively binding on Parties to the Convention), and rooibos tea, like coffee and tobacco, had been in the public domain for around 150 years by then.
2. Given that the rooibos industry is no longer involved in *bioprospecting* ^20^, an activity captured under the CBD, but only in *biotrade* ^21^—which is not explicitly part of the CBD and the Nagoya Protocol—why wouId benefit sharing be due?

The following answers were eventually accepted by representatives from the rooibos industry:

First, the commercialization of rooibos was based on the originally shared TK, a scenario already envisioned by the CBD and NEMBA, therefore there was no time limit on the issue of restitution.

Second, NEMBA, the South African Biodiversity Act, is broader than the CBD and the Nagoya Protocol, incorporating in its remit *unchanged* indigenous biological resources such as tea, as long as their use is based on indigenous TK, and as long as there is commercial exploitation. NEMBA defines *bioprospecting* as:

any research on, or development or application of, indigenous biological resources for commercial or industrial exploitation”… “including the utilisation … of any information regarding any traditional uses of indigenous biological resources by indigenous communities.^22^

Hence, any indigenous biological resource, changed or unchanged, that is used commercially and based on TK is subject to benefit sharing. Given that the RBSA was signed under the auspices of the South African Department of Environmental Affairs, rooibos as an unchanged tea as well as an ingredient for health and cosmetic products was covered by the biodiversity legislation.

Third, “countless rooibos advertisements … exploit the image of San and Khoi and their traditional links to rooibos… [Hence,] clearly, there is a case to be made for benefit sharing linked to traditional knowledge.”^23^

Fourth, there is a strong link between bioprospecting as defined in the CBD and rooibos, even though this was not the focus of the benefit sharing agreement under discussion here. Rooibos-related products are available in cosmetics, novel foods, slimming products, extracts, and flavorants.^24^ Whilst rooibos is mostly known as a tea, it currently has in excess of 140 patents pending for its biochemical and health properties. Hence, with benefit sharing payments being levied at the processing level (see below), rooibos resources for uses other than tea are also *de facto* covered by the agreement. Additional protection is derived from rooibos’ geographical indication, which means it has to be grown in the Cederberg area.

The next section outlines the complex and lengthy process from the initial claim by the San that benefit sharing was due through to the RBSA.

## The Process

Through the CBD, “indigenous peoples’ and local communities’ rights to … control over their own TK are receiving greater recognition than at any time in recent history.”^25^ The RBSA is an example of indigenous peoples taking such control over their TK.

In 2010, the South African San Council initiated steps to challenge the South African rooibos industry over their use of traditional knowledge in relation to rooibos. The process is summarized in Table 1.^26^

**Table 1.** Rooibos Benefit Sharing Agreement—the Process

Date	Events and Actions
**09/2010**	San Council letter to South African Minister of Environmental Affairs to note noncompliance of rooibos industry with National Environmental Management Biodiversity Act.
**11/2011**	First meeting between San Council, South African Department of Environmental Affairs (DEA),^27^ Rooibos Council and Honeybush Tea Association. Representatives of Rooibos Council claim that the NEMBA does not apply to the rooibos industry.
**01/2012**	San Council receives letter from DEA Minister approving the process to explore the rights of the San under NEMBA in relation to rooibos.
**05/2012**	No further communication following the January letter from DEA. San Council letter to DEA in May threatening legal action if there was no significant progress.
**06/2012**	DEA provides action plan to San Council.
**07/2012**	San case presented to Rooibos and Honeybush Associations at meeting organized by DEA. Representative of Rooibos Council described the TK case as “vague.”
**08/2012**	San Council hold discussions with the Khoi leadership, and invites them to join as equal partners in the benefit sharing case. The two legal teams are *Chennells Albertyn* for the San, and *Natural Justice* for the Khoi.
**2012**	Concerns expressed to the DEA by Heiveld Cooperative in Nieuwoudville^28^ that the “true knowledge bearers” of rooibos, namely the small farming communities in the Cederberg mountains, had been excluded from discussions. San and Khoi commenced process to ensure inclusion.
**11/2012**	First full meeting between all stakeholders of the rooibos and honeybush industry, as well as Khoi, San, and DEA. Legal representatives present the case of the San and Khoi as TK holders. The official response of the rooibos industry was that it was not convinced by the TK claim, and they saw no reason to negotiate.
**01/2013**	Letter sent to DEA by the combined legal teams setting out the history of attempts to bring the rooibos industry to the table to negotiate, and threatening legal action if the stalemate was not broken.
**07/2013**	Meeting at DEA between DEA personnel, San, Khoi, and respective legal teams to address the deadlock.
**07/2013**	Memorandum of Association, signed between San Council and National Khoi-San Council, establishes both as equal partners in all matters related to the RBSA. (This approach had been informally agreed-to in July 2012).
**2013-2014**	San and Khoi attempt to secure progress with negotiations by holding informal meetings with governmental officials and representatives of the rooibos industry, and addressing letters to DEA as well as other interested parties.
**06/2014**	DEA commissions Traditional Knowledge study (known as *The TK study*) to ascertain whether San and/or Khoi are traditional knowledge holders on rooibos.
**08/2014**	DEA addresses letter to Rooibos Council informing them that they were obliged to negotiate a benefit sharing agreement. Rooibos Council does not respond; elects to await the outcome of the *TK study.*
**10/2014**	DEA receives commissioned expert *TK study* on the rooibos and honeybush species, which fully supports the TK claim of the San and Khoi.
**07/2015**	*TK study* released to rooibos industry, including the conclusion that “there is no evidence to dispute the claim by the San and the Khoi people of South Africa that they are the rightful holders of traditional knowledge associated with Rooibos and Honeybush.”
**09/2015**	Meeting between rooibos and honeybush industry, with San, Khoi, and DEA. Rooibos industry representatives explain that rooibos is required to pass through 10-11 ‘processors,’ before proceeding to distributors and marketers. Parties agree that the negotiation process will focus on rooibos alone rather than rooibos and honeybush. The idea of capturing all rooibos production at the level of processors, namely at the narrowest part of the product cycle, was born.
**12/2015**	Official meeting between all stakeholders opens negotiations. The rooibos industry representatives stated that they reserved their rights on acknowledging of TK, did not accept the outcome of the *TK study*, and intended to fund a further study to possibly counter or oppose the *TK study* approved by the DEA.
**02/2016**	San and Khoi brief counsel in the expectations of having to take their TK claim to court. Teams prepare for litigation in the event that negotiations fail.
**08/2016**	DEA called a formal meeting to initiate “negotiation of benefit sharing for commercial utilisation of rooibos and its associated traditional knowledge.”
**08/2016**	Further meetings include representatives from Wupperthal and Nieuwoudville in the Cederberg mountains, representing the most important rooibos farming communities, who have contributed significant knowledge relevant for the commercialization process.^29^
**11/2016**	Code of conduct for negotiations agreed, including confidentiality clauses. Meetings from this point onwards explore a joint purpose rather than being expressed through pure opposition.
**12/2016**	Discussion of specific issues central to the agreement, such as how to include all rooibos stakeholders, whether a percentage or fixed-price levy is more appropriate, how an agreement affects intellectual property rights, etc.
**02/2017**	Levy model agreed. Levy to be charged at ‘farm gate price’ paid by processors of rooibos, ensuring compliance of all upstream and downstream stakeholders.
**2017**	Much communication takes place between meetings. Financial models, legal arrangements, and competing proposals are shared and debated in private meetings and correspondence.
**05/2017**	Deadlock or stalemate reached. ‘Lockdown’ meeting to ascertain financial details and consequences of different levels of TK levy. Parties not prepared or able to compromise. Appointment of mediators to resolve the deadlock.
**2017-2018**	Over the following two years the parties engage actively and with a growing shared belief that a benefit sharing agreement was possible. Mediation ended. DEA as facilitator remains independent and supportive of the parties.
**11/2018**	Agreement reached in principle that a TK levy of 1.5 percent of ‘farm gate price’ was fair and reasonable to both sides. Related legal matters required further negotiations.
**03/2019**	A full agreement accepted by all parties, with a private signing on March 25, 2019. Present at the signing were the Chairpersons and appointed representatives of the San, the Khoi, the Rooibos Council, and DEA.
**03/2019**	Five suspensive conditions were required to be met, before the agreement was final and binding under NEMBA. 1. A benefit sharing agreement in the form prescribed by the Biodiversity Act must be concluded (see last entry under 03/2019). 2. Approval of the benefit sharing agreement by the Minister. 3. A written undertaking from DEA to pay the financial cost of administering the annual TK levy together with all audited financial reports. 4. Finalization and registration of the respective trust deeds of the San Council and the National Khoi-San Council providing copies to DEA considering comments from the South African Rooibos Council (the administrative arm of the rooibos industry for this agreement). 5. A Standard Operating Procedure for collecting the TK levy agreed by the parties.
**2019**	The agreement was launched November, 1, 2019 at !Khwa ttu, a San cultural heritage and training center near Cape Town, by Minister Barbara Creecy.

It was accepted that due to the ground-breaking nature of the agreement, it would need to be fully reviewed in a year. It was also agreed that the launch would need to take into account the international relevance of the first comprehensive industry-wide benefit sharing agreement to be formed in accordance with the CBD and Nagoya Protocol.

## The Rooibos Benefit Sharing Agreement

Benefit sharing agreements in South Africa are governed by contract law, and thus parties can agree terms of their own choosing, guided by and within the framework set by NEMBA. The process of negotiating benefit sharing agreements under NEMBA is required to be supervised by the relevant ministry, the South African Department of Environmental Affairs, and finally approved by the Minister.

The full title of the RBSA^30^ is:

BENEFIT SHARING AGREEMENT In accordance with the National Environmental Management: Biodiversity Act 10 of 2004, based upon the principles contained in the 2010 Nagoya Protocol on Access to Genetic Resources and the Fair and Equitable Sharing of Benefits Arising from their Utilisation on Biological Diversity adopted in the International Convention on Biological Diversity (1992).

**Figure 2.**
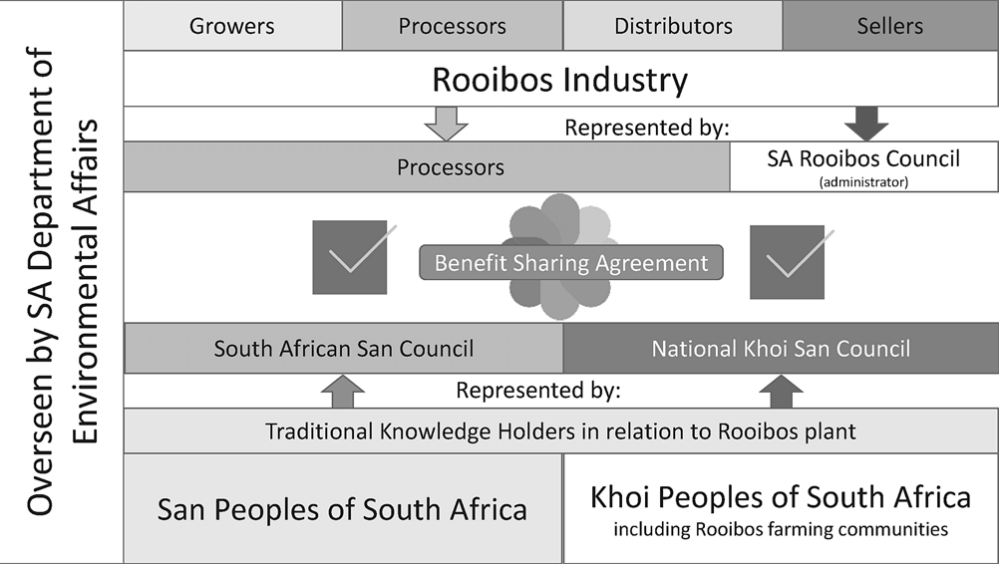
Partners to the Rooibos Benefit Sharing Agreement.

The partners to the agreement are illustrated in Figure 2. The San and Khoi people are recognized in the agreement as the traditional knowledge holders, who are represented by their two Councils. The rooibos industry value chain has four main groups, and in the RBSA, they are represented by one of those groups (the processors) and an administrative council. DEA facilitated and oversaw the benefit sharing negotiations.

Whilst the Nagoya Protocol^31^ provides sample benefits (see Table 2), other benefits can be agreed.

**Table 2.** Overview of Benefit Sharing Examples from Nagoya Protocol

**Monetary benefits**	**Nonmonetary benefits**
Access fees per sample collected	Collaboration in scientific research
Payment of royalties or licence fees	Technology transfer under fair and most favourable terms
Research funding	Institutional capacity-building
Joint ventures	Research directed toward priority needs
Joint ownership of intellectual property rights	Food and livelihood security benefit

The RBSA includes both monetary benefits and the potential future specification of nonmonetary benefits.

**Figure 3.**
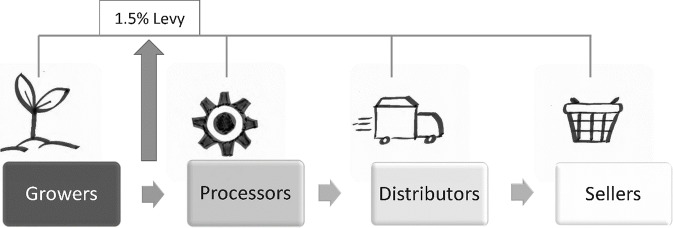
Point in the Value Chain for Levy.

The monetary benefit of the RBSA is an annual levy of 1.5 percent on the ‘farm gate price.’ The farm gate price is what processors (those who clean, dry, ferment, pasteurize, extract, etc.) pay for unprocessed rooibos. Figure 3 summarizes the rooibos value chain and shows at which point in the chain the levy is placed.

The levy is paid by rooibos processors at the end of each financial year to DEA and then paid by DEA into two trust accounts, one created for the San, one created for the Khoi. By signing the RBSA, the rooibos industry also committed to exploring nonmonetary benefits for the TK holders. Whilst these have not yet been detailed, the RBSA suggests some examples, namely: “the creation of employment opportunities, bursaries, development schemes, mentoring and facilitation of livelihood creation, and other ways of delivering alternative forms of benefits to the two traditional knowledge holding communities.” Each community receives 50 percent of the total payment.

The levy which goes to the Khoi people is to be shared equitably with indigenous farming communities in the Cederberg mountains (see ‘Challenges’ section for the reasons why this was agreed).

The RBSA is anticipated as a “long-term partnership… based upon utmost good faith, where each Party strives for and contributes towards the success and good fortune of the other.”^32^ The good faith element includes:

• confidence expressed in the RBSA that the TK holders “warrant and undertake that they represent and incorporate all groups and communities currently known to them… which may … hold TK [traditional knowledge] in respect of Rooibos.”
• recognition that the “economic viability of the Processers” is important and needs to be taken into account in any potential review of the levy.
• the commitment by the TK holders to “generally support… marketing and publicity efforts aimed at increasing the success of Rooibos products worldwide.”^33^

Thus, the aim of the RBSA is to achieve optimal fairness between partners and:

to provide the maximum possible sharing of Rooibos benefits as required by the Biodiversity Act, whilst ensuring that the Annual Levy is sustainable and not damaging to the Rooibos industry, yet represent fair and equitable sharing of benefits as outlined in Nagoya Protocol.^34^

## The challenges

Arriving at a complex industry-wide benefit sharing agreement was a highly challenging task, as the nine year timeline above indicates. Each of the main challenges below needed to be resolved before the negotiations could continue.

### Representation

One prominent reason why benefit sharing negotiations can fail is the criticism that local communities are not fairly represented amongst negotiators. In this regard, the most famous case to date is the Maya ICBG (International Co-operative Biodiversity Groups) Project in Chiapas, Mexico.^35^ “The failure of the Maya ICBG was due largely to the lack …. of adequate representation.”^36^

Having successfully negotiated the *Hoodia* benefit sharing agreement,^37^ and the *Sceletium* benefit sharing agreement (see Box 1), the representative structures of the San community had already been tested and used successfully.^38^ ^,^^39^ Particular challenges that were overcome for the earlier benefit sharing agreements were, in the *Hoodia* case, the transnational nature of San residence in Southern Africa, and, in the *Sceletium* case, the involvement of non-San informants regarding the medicinal use of plants. Yet, the rooibos case was even more complex, because:

• an entirely separate indigenous group, the Khoi, also held traditional knowledge of rooibos,
• significant progress to enable commercialization of rooibos was made by another group, namely, small-scale farmers in the Cederberg mountains, and
• there was no precedent worldwide for a comprehensive, industry-wide benefit sharing agreement.

With the addition of a serious lack of resources, these challenges are discussed below.

#### San and Khoi alliance.

The San and the Khoi peoples of South Africa are both regarded as indigenous communities in Southern Africa.^40^ Whilst anthropologists and archaeologists present a range of different theories, the ancient rock art of Southern Africa is attributed to the San, who are predominantly of hunter gatherer origin, whilst the Khoi pastoralists entered South Africa later.^41^ The *TK study* commissioned by DEA (see timeline) confirmed that both groups held traditional knowledge in relation to rooibos.

Whilst the San Council *started* the benefit sharing process, it was clear to the San leadership that the Khoi held a similar claim and that both groups needed to be included in any benefit sharing negotiations. The San leadership was also aware of the Chiapas (Maya ICBG) case, where benefit sharing negotiations had collapsed because agreement amongst several groups could not be achieved. Hence, being conscious of the high value of unity, in August 2012 the San Council invited the Khoi leadership to discuss a possible joint approach in relation to rooibos. A memorandum of understanding^42^ (MOU) between the two indigenous groups was concluded, committing them to form a joint negotiating body in all matters relating to the rooibos, honeybush, buchu, and hoodia plant species.^43^ It further stated that “any benefits received will be divided equally between them.” This MOU ensured that the two important issues of representation and division of benefits were dealt with early on, and that the parties could henceforth concentrate on negotiating the best possible outcome as equal partners.

Considering the above, the RBSA can therefore be regarded as a success story for Article 11 of the Nagoya Protocol, which states that:

Where the same traditional knowledge associated with genetic resources is shared by one or more indigenous and local communities in several Parties, those Parties shall endeavour to cooperate, as appropriate, with the involvement of the indigenous and local communities concerned, with a view to implementing the objective of this Protocol.^44^

#### Rooibos farming communities.

Rooibos is farmed only in the Cederberg mountains north-east of Cape Town (see Figure 1), where many descendants of early Khoi live as small-scale farmers and farm workers. The Cederberg mountains are also the region for which a geographical indication on rooibos was obtained in 2014.

In and around the Cederberg towns of Wupperthal in the south, and Nieuwoudville in the north, rooibos is regarded as a way of life.^45^ In these locations, an estimated 75 percent of local income is derived from rooibos tea,^46^ whilst up to 90 percent of the local population (small-scale farmers and workers) were discriminated against during apartheid. Hence, “the geographical and political backdrop to the rooibos industry is one of dispossession and adversity.”^47^

A central tension [in any rooibos benefit sharing] is the balance between achieving historical and restorative justice for the San and Khoi and recognizing the many others who have provided knowledge towards the success of the rooibos industry.^48^

That rooibos is sold successfully all over the world as a tea is *also*, Wynberg argues, reliant upon contributions from inhabitants of the Cederberg mountains.

Such contributions … [include] momentous discoveries of individuals such as Tryntjie Swarts, who located the “golden nests” of rooibos seed in the 1920s and thus facilitated the industry's expansion; [and] Annekie Theron, who accidentally discovered in 1968 that rooibos had a soothing effect on her hyper-allergic baby, leading to a dramatic increase in demand for rooibos.^49^

Against this backdrop, the National Khoi Council began consultations in the Cederberg Mountains and by 2013 elected representation of the Cederberg farming communities had been secured as part of the Khoi delegation of the benefit sharing negotiations.

As a result of the presence of the farming communities in the Khoi negotiation team, the RBSA includes a clause that the benefits from the agreement which are to go to the Khoi Trust, will be shared with the small-scale farming communities in the Cederberg Mountains.

### Lack of resources

A common theme raised by indigenous organizations advocating for the various rights created in both international and domestic legislation is the massive and inherent power differential between them and their more established negotiating opponents.^50^

In this case, the rooibos industry is commercially successful, with modern means of communication and the capacity to rely upon legal advice from professional accountants, as well as a leading international commercial law firm. The indigenous peoples by contrast relied on voluntary organizations representing the poorest of South African communities, with no immediate funding for legal representation and a lack of access to modern means of communication.

Whilst DEA has fulfilled its facilitative role in funding travel and logistics for the plenary meetings since 2012, both the San and Khoi were challenged by their lack of funding and capacity. The South African San Council had been functioning on a skeleton budget since 2001 that enabled them to employ only one or, at the most, two staff supporting a volunteer council. They had no additional funds to pay for community meetings, travel, or legal support. The lawyer representing the San interests did most of the work on the case pro bono. The National Khoi-San Council, despite having been formed by the government in 1999, had never been allocated funding, and it relied solely upon funds raised by its legal representatives, the environmental rights NGO *Natural Justice.* Legal and intellectual property advice for the TK holders was therefore reliant upon charity, as well as various international supporters of indigenous rights.

### Complex, industry-wide Benefit Sharing Agreement

To date, no other comprehensive, industry-wide benefit sharing agreement has been concluded under the CBD and the Nagoya Protocol. Hence, there was no precedent from which the parties to the RBSA could learn.

Throughout the negotiations, the central question remained the issue of what constitutes a *fair* levy, i.e., both what the industry could afford, and what represents fair restitution for the holders of TK.

Numerous proposals were made as to how the large and complex rooibos industry could be covered by a single agreement with one point of collection for monetary benefits. Several financial models, legal arrangements and competing proposals were shared in private meetings and correspondence.

A mediation process was required to assist progress toward the final agreement that a 1.5 percent levy would be collected by processors of rooibos and shared with the TK holders (see Figure 3). As all rooibos is processed before it is either exported or converted into tea and other products, this model enables the cost of the levy to be spread and shared both ‘upstream’ to the growers, as well as ‘downstream’ to the distributors and sellers.

The groups involved in the RBSA overcame significant obstacles which had led to the collapse of similar benefit sharing agreements in other geographic regions. Of particular importance was the aforementioned unity amongst the San and Khoi as TK holders, the recognition of the contributions of small-scale farmers, and the willingness of the rooibos industry to cover all rooibos production and sale with one benefit sharing agreement.

## The relationship with the San Code of Research Ethics

Indigenous peoples are frequently considered to epitomize vulnerable populations in need of protection from exploitation^51^ ^,^^52^ ^,^^53^ ^,^^54^ whereby being ‘vulnerable’ can be defined as:

[facing] a significant probability of incurring an identifiable harm while substantially lacking ability and/or means to protect oneself.^55^

Being exploited in research, or biotrade and bioprospecting, is a serious, identifiable harm. Taking the San community as an example,^56^ deeply respected San community leaders have repeatedly expressed their concerns about such exploitation and have:

In recent years …, with increasing confidence, arrived at the conclusion that most … research on their communities was neither requested, nor useful, nor protected in any meaningful way. In many cases dissatisfaction if not actual harm was the result.^57^

The effects of collective trauma (e.g., past genocide), loss of traditional lands, extreme poverty, lack of access to education, as well as lack of funds to employ outsiders, all meant “that the protection of … [San] traditional knowledge was precarious.”^58^

Yet, the San community have managed what no other indigenous community has done in Africa before, i.e., they were the first indigenous community on the continent to issue their own code of research ethics.^59^ The San Code of Research Ethics (2017) is built around four substantial moral values, and also requires due process. After a range of consultations, the authors of the San Code were clear that they wanted respect, honesty, fairness, and care^60^ from researchers and those using their TK. Unusually, and very effectively, the authors decided to add exploitation examples *within* the ethics code. For instance, under fairness, they wrote:

We have encountered lack of justice and fairness in many instances in the past. These include theft of San traditional knowledge by researchers. At the same time, many companies in South Africa and globally are benefitting from our traditional knowledge in sales of indigenous plant varieties without benefit sharing agreements, proving the need for further compliance measures to ensure fairness.^61^

For rooibos, the RBSA ensures that the sales of indigenous plants based on TK no longer occurs without benefit sharing, thereby satisfying the moral value of fairness. Table 3 shows how respect, honesty, fairness, and care are prominently worked into the RBSA, which was informed in the crucial last years of negotiations by the San Code of Research Ethics. The examples given are not exhaustive.

**Table 3.** Respect, honesty, fairness, and care in the Rooibos Benefit Sharing Agreement (RBSA 2019)

Respect	Honesty	Fairness	Care
“The Parties … acknowledged the San and the Khoi-Khoi’s as being the holders of TK.”	“The San Council and the National Khoi-San Council undertake to provide annual reports of its audited financial statements to DEA.”	“The Processors hereby commit to exploring non-monetary benefits … for the communities.”	“Each Party strives for and contributes towards the success and good fortune of the other.”
“This agreement [is based] upon the utmost good faith.”	“The audit certificate of each Processor shall indicate the volumes of Rooibos purchased.”	“The TK holders… undertake to ensure that the benefits… are fairly distributed to the beneficiaries including the Rooibos Indigenous Farming Communities.”	“Should a dispute … arise between the Parties …, the Parties commit themselves to a sincere attempt to resolve the dispute.”

One could argue that the main test for respect, honesty, fairness, and care is still to come, namely in the distribution of funding to local communities. Again, taking the San community as an example, the Andries Steenkamp^62^ Benefit Sharing Trust, which will be responsible for distributing funding to the San community, is clearly linked to the values of the San Code of Research Ethics. Its main principle is as follows:

The basis of all operations, functions and administration of this Trust will strictly be to assist San communities in their endeavours to protect their traditional knowledge and related biodiversity, to protect their cultural heritage, to advance their education and development and to improve their livelihoods; and doing so with respect, honesty, fairness and care.^63^

In addition, the value of honesty is particularly strong in the provision that “the Trust and subsidiaries must be audited … on an annual basis,” whilst the values of care and fairness are particularly clear in the provision that “the Board of Trustees will not be paid any form of remuneration … as this Trust is run to the benefit of communities.”

## Policy implications and outlook

The RBSA, based on respect, honesty, fairness, and care, is a major achievement.

• Financially, it is the biggest benefit sharing agreement between industry and TK holders since the adoption of the CBD more than a quarter of a century ago.
• It is the first comprehensive, industry-wide agreement to be formed in accordance with biodiversity legislation, which implements the CBD and Nagoya Protocol.
• The benefit sharing agreement is based on a well-established product, not a patent. Hence, on this occasion, it is already clear that a significant funding stream will become available to TK holders.

The government ministry DEA played an active facilitating role in encouraging the parties to reach a settlement, without losing objectivity or the trust of the parties. This active government support played a significant role in enabling resolution and signing of the RBSA.

With no precedent in the benefit sharing world, the RBSA stands as a concrete example of the ‘art of the possible.’ It serves to confirm that such agreements can be concluded in support of indigenous communities, industry, and governments implementing the CBD and Nagoya Protocol. It offers lessons and inspiration to indigenous communities, governments, and industry sectors both within Africa and other regions around the world. The mere fact that it has been successfully concluded should serve as confirmation that benefit sharing agreements of such scale and complexity are possible.

Global, regional and national policy makers would do well to pay heed to the RBSA. While the rooibos case is unique in a number of aspects, the experience offers several transferable insights regarding elements of success, including: patience; incrementalism; honesty, trust; genuine dialogue; strong legal support; a shared recognition that a fair, win-win deal is possible; government facilitation and leadership; and unity amongst indigenous peoples. Such ingredients of success can, of course, apply well beyond southern Africa.

Nevertheless, one could argue that the main challenges for the RBSA partners are still to come, as they implement the agreement to benefit the guardians of TK with respect, honesty, fairness, and care.

## Notes

1. Convention on Biological Diversity 1992; available at https://www.cbd.int/doc/legal/cbd-en.pdf (last accessed 17 Aug 2019).

2. Schroeder D, Bisupati B. Ethics, Justice and the Convention on Biological Diversity, United Nations Publication, 2010; available at https://tinyurl.com/y49j2yke (last accessed 17 Aug 2019).

3. Nagoya Protocol 2010; available at https://www.cbd.int/abs/doc/protocol/nagoya-protocol-en.pdf (last accessed 17 Aug 2019).

4. National Environmental Management Biodiversity Act (NEMBA) 2004; available at https://www.environment.gov.za/sites/default/files/legislations/nema_amendment_act10.pdf (last accessed 17 Aug 2019).

5. In 1995, human genetic resources were excluded from the CBD, leaving genetic resources of plant, animal, and microorganism origin, as well as related traditional knowledge within its scope, see Schroeder D . Benefit Sharing – High Time for a Definition. Journal of Medical Ethics 2007;33(4):205–91740061710.1136/jme.2006.016790PMC2652775.

6. For example, the 2016 Swiss Academy of Science Agreement on Access and Benefit-sharing for Academic Research; available at https://tinyurl.com/y53a8zru (last accessed 17 Aug 2019).

7. For example, World Intellectual Property Organization. Model Access and Benefit Sharing Agreement between Australian Government and Access Party; available at https://www.wipo.int/tk/en/databases/contracts/texts/australiaprovider.html (last accessed 17 Aug 2019).

8.

Knight A , Underhill P , Mortensen H , Zhivotovsky L , Lin A , Henn B , Loui D , Ruhlen M , Mountain J . African Y Chromosome and mtDNA Divergence Provides Insight into the History of Click Languages. Current Biology 2003;13(6):464–731264612810.1016/s0960-9822(03)00130-1.

9.

Barnard A . Hunters and Herders of Southern Africa: A comparative Ethnography of the Khoisan Peoples. New York and Cambridge: Cambridge University Press; 1992.

10. The licensing to Pfizer and Unilever was indirect via a UK company called Phytopharm.

11. Additional small-scale benefit sharing agreements were signed by the South African San Council with individual companies, including HG&H (Pty) Ltd (sceletium, see Box 1), Cape Kingdom (buchu), Nestle (rooibos), Puris (Pty) Ltd (buchu) and Zuplex (Pty) Ltd (rooibos, hoodia, buchu, and others).

12. The term ‘Khoi-San’ is increasingly used in the public domain as a unifying name for the Khoi or KhoiKhoi, and the San or Bushmen—two distinct groupings in southern Africa. However, this umbrella term is not suitable for discussing the San peoples. The Khoi or KhoiKhoi are regarded as pastoral and of more recent descent than the San, who are descended from hunter-gatherers (see note 9, Barnard 1992).

13. Translational science is the effort of building on basic research leading up to specific products. The term is mostly used in medicine, and is also referred to as ‘bench to bedside’ research, see Goldblatt E , Lee W . From bench to bedside: The growing use of translational research in cancer medicine. American Journal of Translational Research 2010;2(1):1–18.20182579PMC2826819

14. San Code of Research Ethics 2017; available at http://www.globalcodeofconduct.org/affiliated-codes/ (last accessed 17 Aug 2019).

15. Whilst the health properties of rooibos claimed by the San and Khoi are not proven, there are at least 141 patents pending or registered in relation to various health applications (see Wynberg R . Making sense of access and benefit sharing in the rooibos industry: Towards a holistic, just and sustainable framing. South African Journal of Botany 2017;110:39–51).

16. Siyanda Samahlubi Consulting for DEA. Traditional Knowledge Associated with Rooibos and honeybush Species in South Africa, October 2014.

17. South African Rooibos Industry. South African Rooibos Industry Report for Benefit Sharing Negotiations as made available to legal teams, 2017.

18. World Intellectual Property Organization. Rooibos - Disputing a Name, Developing a Geographical Indication, 2018; available at https://www.wipo.int/ipadvantage/en/details.jsp?id=2691 (last accessed 17 Aug 2019).

19. First source: Rooibos Council. Rooibos Industry Fact Sheet 2018; available at https://sarooibos.co.za/wp/wp-content/uploads/2018/08/SARC-2018-Fact-Sheet-1.pdf (last accessed 17 Aug 2019). Second source: see note 15, Wynberg 2017. Third source: Joubert E , de Beer D . Rooibos (Aspalathus linearis) beyond the farm gate: From herbal tea to potential phytopharmaceutical. South African Journal of Botany 2011;77(4):869–86.

20. According to NEMBA, bioprospecting means “any research on, or development or application of, indigenous biological resources for commercial or industrial exploitation” (see note 4, NEMBA 2004).

21. Biotrade means “the current buying and selling of milled, powdered, dried, sliced or extract of indigenous genetic and biological resources for further commercial exploitation” (see UNCTAD. Facilitating BioTrade in a Challenging Access and Benefit Sharing Environment. UNCTAD/WEB/DITC/TED/2016/4:11; available at https://unctad.org/en/PublicationsLibrary/webditcted2016d4_en.pdf (last accessed 17 Aug 2019).

22. See note 4, NEMBA 2004, at 13.

23. See note 15, Wynberg 2017.

24. Wynberg R. Rooibos: a testing ground for ABS in South Africa. University of Cape Town, Voices for Biojustice, 2018; available at http://bio-economy.org.za/wp-content/uploads/2018/04/Rooibos-Policy-Brief-FINAL-1.pdf (last accessed 17 Aug 2019).

25.

Laird S , ed. Biodiversity and Traditional Knowledge: Equitable Partnerships in Practice. London: Earthscan; 2002 , at 417.

26. The information in the table was provided by those co-authors who were part of the process, the lawyer acting on behalf of the San (RC) and the San Leadership (CL, LS).

27. In June 2019, DEA became DEFF (Department of Environment, Fisheries and Forestry). The earlier name, DEA, will be used throughout this publication, as it covers the nine years preceding the signing of the Rooibos Agreement in March 2019.

28. The Heiveld Coooperative (http://www.heiveld.co.za/index.html) was formed in 2000 to represent members from the local rooibos farming community in the region of the Cederberg known as the Suid Bokkeveld.

29. See note 15, Wynberg 2017.

30. The Rooibos Benefit Sharing Agreement (RBSA, 2019) is currently only available to the negotiating partners.

31. See note 3, Nagoya Protocol 2010.

32. See note 30, RBSA 2019.

33. See note 30, RBSA 2019.

34. See note 30, RBSA 2019.

35.

Berlin B , Berlin E . Community Autonomy and the Maya ICBG Project in Chiapas, Mexico: How a Bioprospecting Project that Should Have Succeeded Failed. Human Organization 2004;63(4):472–86.

36.

Feinholz-Klip D , Barrios L , Cook Lucas J . The Limitations of Good Intent: Problems of Representation and Informed Consent in the Maya ICBG Project in Chiapas, Mexico In: Wynberg R , Schroeder D , Chennells R . Indigenous Peoples, Consent and Benefit Sharing – Lessons Learned from the San Hoodia Case. New York and London: Springer; 2009, 315–31, at 315.

37. See note 36, Wynberg 2009.

38. Chennells R, Haraseb V, Ngakaeaja M. Speaking for the San: Challenges for Representative Institutions. In Wynberg 2009 (see note 37), at 165–89.

39. Chennells R, Schroeder D. The San Code of Research Ethics - Its Origins and History, Report for the South African San Council and the TRUST project, 2019; available at http://www.globalcodeofconduct.org/wp-content/uploads/2019/02/SanCodeHistory.pdf (last accessed 17 Aug 2019).

40. Stavenhagen R. Human rights and indigenous issues - Report of the Special Rapporteur on the situation of human rights and fundamental freedoms of indigenous people. UN Economic and Social Council, 2005 E/CN.4/2006/78/Add.2.

41. “For thousands of generations, San populations lived by hunting and gathering as the sole occupants of Southern Africa,” Hitchcock R, Biesele M, Ikeya K. Updating the San: Image and Reality of an African People in the Twenty-first Century. *Senri Ethnological Studies* 2006;70.

42. San and Khoi Memorandum of Association signed on July 18 2013, which committed them to joint negotiations regarding the rooibos, honeybush, hoodia, and buchu plant species.

43. Honeybush was also investigated by the TK report commissioned by the DEA to investigate TK. On receipt of the report, the San and Khoi commenced negotiations on Rooibos first.

44. See note 3, Nagoya Protocol 2010.

45.

Ives S . Farming the South African ‘bush’: Ecologies of belonging and exclusion in rooibos tea. American Ethnologist 2014;41(4):698–713 .

46. Wynberg R, Custers S. Determining a Fair Price and Equitable Benefit for Small-Scale Rooibos Tea Producers. An Analysis of the Costing and Pricing of Small-Scale Production of Organic and Fair Trade Rooibos, and Benefit Flows along the Rooibos Value Chain, 2005. Report Prepared for Fair Trade Assistance, Netherlands.

47. See note 15, Wynberg 2017.

48. See note 15, Wynberg 2017.

49. See note 15, Wynberg 2017.

50. See note 36, Wynberg 2009.

51. See note 38, Chennells 2009.

52.

Chennells R . Vulnerability and Indigenous Communities: Are the San of South Africa a Vulnerable People? Cambridge Quarterly of Healthcare Ethics 2009;18(2):147–541925056810.1017/S0963180109090240.

53.

Weijer C . Protecting Communities in Research: Philosophical and Pragmatic Challenges. Cambridge Quarterly of Healthcare Ethics 1999;8(4):503–1310.1017/s096318019900412010513308.

54.

Weijer C , Golds G , Emanuel E . Protecting communities in research: Current guidelines and limits of extrapolation. Nature Genetics 1999;23:275–80.1054594610.1038/15455

55.

Schroeder D , Gefenas E . Vulnerability – Too Vague and Too Broad? Cambridge Quarterly of Healthcare Ethics 2009;18(2):113–21.1925056410.1017/S0963180109090203

56. Given that the San leadership co-authored this paper, the San example is taken here. The Khoi leadership or the indigenous farming communities would be able to give other examples from their communities. The principle about the vulnerability to exploitation would, however, be the same.

57. See note 39, Chennells 2019.

58. See note 52, Chennells 2009.

59. Callaway E. South Africa’s San people issue ethics code to scientists. In: *Nature | News*, 20 March 2017; available at https://www.nature.com/news/south-africa-s-san-people-issue-ethics-code-to-scientists-1.21684 (last accessed 17 Aug 2019).

60. For a definition of the values, see Schroeder D , Chatfield K , Chennells R , Singh M , Herissone-Kelly P . Equitable Research Partnerships - A Global Code of Conduct to Counter Ethics Dumping. New York and London: Springer; 2009.

61. See note 14, San Code of Research Ethics 2017.

62. Andries Steenkamp, a former chairperson of the South African San Council, was a leader of unique personal integrity who “exuded an air of confidence and open curiosity, quick to understand and appreciate the persons across the table, and slow to take personal offence” (see note 39, Chennells 2019). Andries was one of the main negotiators of the Rooibos agreement until his early death in 2016. In his honor, his name was chosen for the RBSA trust that will benefit the San.

63. All quotes from the Andries Steenkamp Benefit Sharing Trust were obtained from the South African San Council. The deeds for the trust are not publicly available.

